# Policy disparities in response to the first wave of COVID-19 between China and Germany

**DOI:** 10.1186/s12939-021-01424-3

**Published:** 2021-03-25

**Authors:** Yuyao Zhang, Leiyu Shi, Haiqian Chen, Xiaohan Wang, Gang Sun

**Affiliations:** 1 Department of Health Management, School of Health Management, Southern Medical University, Guangzhou, Guangdong 510515 P. R. China; 2 Department of Health Policy and Management, Bloomberg School of Public Health, Johns Hopkins University, Baltimore, MD 21205 USA

**Keywords:** Global health equity, COVID-19, China, Germany, Nonpharmaceutical intervention

## Abstract

**Objective:**

Our research summarized policy disparities in response to the first wave of COVID-19 between China and Germany. We look forward to providing policy experience for other countries still in severe epidemics.

**Methods:**

We analyzed data provided by National Health Commission of the People’s Republic of China and Johns Hopkins University Coronavirus Resource Center for the period 10 January 2020 to 25 May 252,020. We used generalized linear model to evaluate the associations between the main control policies and the number of confirmed cases and the policy disparities in response to the first wave of COVID-19 between China and Germany.

**Results:**

The generalized linear models show that the following factors influence the cumulative number of confirmed cases in China: the Joint Prevention and Control Mechanism; locking down the worst-hit areas; the highest level response to public health emergencies; the expansion of medical insurance coverage to suspected patients; makeshift hospitals; residential closed management; counterpart assistance. The following factors influence the cumulative number of confirmed cases in Germany: the Novel Coronavirus Crisis Command; large gathering cancelled; real-time COVID-19 risk assessment; the medical emergency plan; schools closure; restrictions on the import of overseas epidemics; the no-contact protocol.

**Conclusions:**

There are two differences between China and Germany in non-pharmaceutical interventions: China adopted the blocking strategy, and Germany adopted the first mitigation and then blocking strategy; China’s goal is to eliminate the virus, and Germany’s goal is to protect high-risk groups to reduce losses. At the same time, the policies implemented by the two countries have similarities: strict blockade is a key measure to control the source of infection, and improving medical response capabilities is an important way to reduce mortality.

## Introduction

Since December 2019, the new coronavirus (SARS-CoV-2) has spread rapidly around the world, with fast spreading speed, wide range of infection, and difficult prevention and control, which has brought a huge impact to the whole world. The World Bank’s projections point to the deepest global recession since World War 2, with millions of people falling into unemployment and poverty [1]. By early November 2020, over 46 million confirmed cases of COVID-19 had been reported worldwide, with more than 1 million deaths [2]. This death toll continues to rise rapidly each day. More than 450,000 new cases have been confirmed in a single day worldwide, and European countries have also officially suffered the second wave of COVID-19. The number of daily new cases in many European countries has exceeded 10,000. The World Health Organization believes that the “epicenter” of the current global epidemic is Europe. Since there is no vaccine or specific treatment for COVID-19, non-pharmacological interventions are particularly important.

On December 31, 2019, Health Commission of Wuhan City reported that 27 cases of viral pneumonia had been diagnosed. On January 9, 2020, the Chinese health expert team identified the virus as a new type of coronavirus, which has a very fast transmission speed. The Chinese government resolutely adopted a series of prevention and control policies to contain the spread of COVID-19. On May 22, 2020, the number of new confirmed cases in China was zero for the first time, and since April 15, the number of new deaths has been zero [3]. This has proved that China’s non-pharmaceutical interventions adopted in the first wave of COVID-19 are effective. In early March 2020, Europe became the epicenter of the COVID-19 pandemic, with more cases and deaths reported than in all other countries (excluding China) combined [4]. In Germany, the first case was recorded on 27 January 2020 in Bavaria [5]. In early March, the cumulative number of confirmed cases in Germany passed 100. A week later, more than 1000 cases were confirmed, and by late March it had passed 10,000 [2]. COVID-19 has spread rapidly, and the prevention and control of SARS-CoV-2 officially kicked off in Germany. In late April, with the joint efforts of the German government and the public, the number of new confirmed cases fell to about 1000, and the number of new deaths gradually dropped to double digits [2]. It can be seen that the non-pharmaceutical interventions implemented by the German government have also played an important role.

We have systematically summarized and quantitatively analyzed the prevention and control policies adopted by China and Germany in the first wave of the global COVID-19 epidemic, and also compared the similarities and differences of non-pharmaceutical interventions by the two governments. We hope to provide policy experience for other countries and regions that are experiencing the second wave of COVID-19.

## Methods

The data are all from the epidemic information released by the National Health Commission of the People’s Republic of China [3] and Johns Hopkins University Coronavirus Resource Center [2]. Data indicators include cumulative confirmed cases, cumulative deaths, daily new cases and daily deaths.

As of May 1, 2020, all Chinese provinces have lifted the first-level public health emergency response (the highest level response) and gradually resume normal life [6]. Since April 20, Germany began to gradually loosen restrictions and return to normalization [7]. We selected the period from the appearance of the first cases to the gradual return to daily life in the two countries.

## Results

### Major non-pharmacological interventions in China

As showed in Table 1,from 10 January to 25 May 2020, COVID-19 outbreak has led the Chinese government to enact the following policies aimed to control the spread of the disease: the Joint Prevention and Control Mechanism; locking down the worst-hit areas; the highest level response to public health emergencies; the expansion of medical insurance coverage to suspected patients; makeshift hospitals; residential closed management; counterpart assistance.

**Table 1:** Seven non-drug intervention policies in China

SN	Date	Policy	Key elements
1	Jan 20	Establishing the Joint Prevention and Control Mechanism	The National Health Commission led the establishment of a joint prevention and control mechanism composed of 32 departments to cope with COVID-19 epidemic, with working groups such as epidemic prevention and control, medical treatment, scientific research, publicity, foreign affairs, logistics support, and forward work.
2	Jan 23	Locking down the worst-hit areas	The government first imposed a lockdown on Wuhan, banned travel to and from Wuhan, suspended all public transportation services in the city, and eventually blocked the entire Hubei Province.
3	Jan 25	Initiating the first-level (highest level) response to public health emergencies	All 30 provinces with confirmed cases initiated the first-level response to public health emergencies, China entered a highly alert state of the national epidemic.
4	Jan 27	Expanding medical insurance coverage to suspected patients	Expanding the coverage of the previous comprehensive guarantee policy for diagnosed patients to suspected patients who meet the diagnosis and treatment guidelines of National Health Commission.
5	Feb 4	Activating makeshift hospitals	Patients with severe to critical COVID-19 received care in Huoshenshan hospital and Leishenshan hospital. Patients with mild to moderate COVID-19 who met additional admission criteria were isolated and treated in mobile cabin hospitals.
6	Feb 10	Residential area closed management	Following the closed management of Wuhan’s residential areas, community grid and digital management were implemented nationwide. Lockdown management was conducted in the building units of confirmed or suspected COVID-19 patients.
7	Feb 12	Counterpart medical assistance	Through “One Province Supports One City”, the entire country supported the epidemic prevention and control in Hubei Province.

### Major non-pharmacological interventions in Germany

From 27 January to 25 May 2020, the surge in the number of confirmed COVID-19 has prompted the German government to develop the following non-pharmacological policies (Table 2): the Novel Coronavirus Crisis Command; large gathering cancelled or postponed; real-time COVID-19 risk assessment; the medical emergency plan; schools closure; restrictions on the import of overseas epidemics; the no-contact protocol.

**Table 2:** Seven non-drug intervention policies in Germany

SN	Date	Policy	Key elements
1	Feb 27	Establishing the Novel Coronavirus Crisis Command	The Ministry of Health and the Ministry of the Interior announced the establishment of a federal-level epidemic response headquarters, and developed a series of prevention and control measures based on the assessment of the German Federal Center for Disease Control.
2	Feb 28	Postponing or cancelling public events	From March to early April, nearly 10 major fairs cancelled or postponed, religious groups banned worship activities, and many professional sports events cancelled.
3	Mar 2	Raising the level of risk assessment for COVID-19	On 2 March, the Robert Koch Institute announced that the risk assessment on the health of the German population for COVID-19 was raised from “low to medium” to “medium”, and on 17 March, the risk assessment level was raised to “high”.
4	Mar 4	Initiating a medical emergency plan	The federal government and the state government reached an agreement to double the number of intensive care beds as soon as possible and called on hospitals to increase the number of beds.
5	Mar 12	Closing schools	Schools and childcare facilities were closed, and the government issued recommendations for social isolation.
6	Mar 15	Controlling the import of overseas cases	The government strengthened border controls and no longer allowed travel through Germany. The EU imposed a 30-day limit on entry for non-EU countries’ citizens.
7	Mar 22	Promulgating the no-contact protocol	The federal government and the state government reached a no-contact protocol: people are required to maintain at least 1.5 m away from each other. It is forbidden to hold large-scale gatherings and carnivals in public places and private apartments.

### Epidemiological timeline of the first COVID-19 wave in China and Germany

**Fig. 1:**
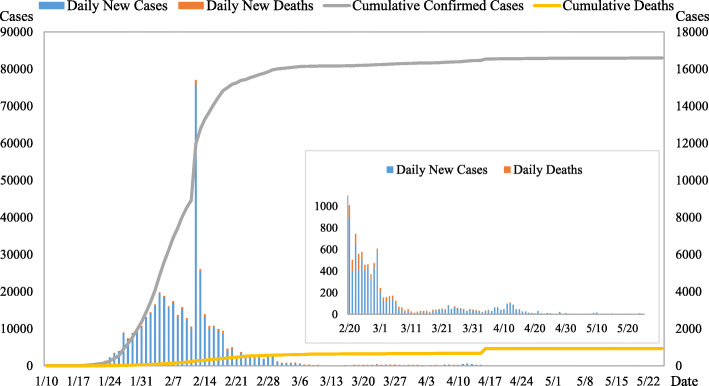
Epidemiological timeline of the first COVID-19 wave in China. Note: Cumulative confirmed cases and the cumulative deaths refers to main axis (left). Daily new cases and daily new deaths refers to secondary axis (right)

Figure 1 shows the development trend of China’s cumulative confirmed cases, cumulative deaths, newly confirmed cases, and new deaths from January 10 to May 25, 2020. Since February 18, the cumulative confirmed case curve has gradually slowed down, and the cumulative death case curve has fluctuated slightly in February and March. The number of newly confirmed cases increased significantly from the end of January to the end of February. At the beginning of March, the number of newly diagnosed cases was less than 200, and the number of new deaths has dropped to zero since late April.

**Fig. 2:**
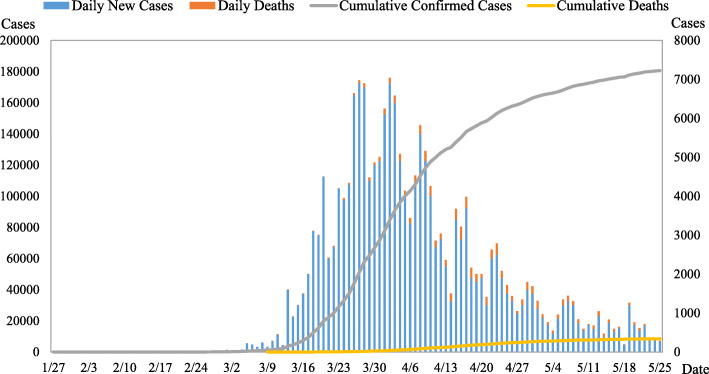
Epidemiological timeline of the first COVID-19 wave in Germany. Note: Cumulative confirmed cases and the cumulative deaths refers to main axis (left). Daily new cases and daily new deaths refers to secondary axis (right)

Figure 2 shows the development trend of the cumulative confirmed cases, cumulative deaths, newly confirmed cases, and new deaths in Germany from January 27 to May 25, 2020. The cumulative curve of confirmed cases fluctuates greatly, with a rapid increase since late March and a gentle trend at the end of April. The cumulative death case curve has fluctuated to a certain extent since late April. The number of newly confirmed cases has increased rapidly since late March, and has declined in late April. The number of new deaths was higher in April.

### Associations between non-pharmaceutical interventions and cumulative number of COVID-19 confirmed cases

Data from China: The cumulative number of confirmed cases is used as the dependent variable Y, and seven non-pharmaceutical indicators in Table 1 are used as independent variables X_1_-X_7_. The results of the generalized linear model (Table 3) show that seven non-drug measures have an impact on the cumulative number of confirmed cases in China.

**Table 3:** Associations between control policies and cumulative number of confirmed cases in China

Parameter	Coefficient	95% CI	P
(Intercept)	11.297	(11.296,11.298)	0.000
Joint Prevention and Control Mechanism	−1.865	(− 1.958,-1.772)	0.000
Locking down the worst-hit areas	−0.892	(−0.961,-0.823)	0.000
The highest level response to public health emergencies	−0.802	(− 0.853,-0.750)	0.000
The expansion of medical insurance coverage to suspected patients	−1.581	(− 1.610,-1.551)	0.000
Makeshift hospitals	−1.044	(−1.052,-1.036)	0.000
Residential closed management	−0.293	(− 0.301,-0.285)	0.000
Counterpart assistance	−0.613	(−0.620,-0.607)	0.000

Data from Germany: The cumulative number of confirmed cases is used as the dependent variable Y, and seven non-pharmaceutical indicators in Table 2 are used as independent variables X_1_-X_7_. The results of the generalized linear model (Table 4) show that seven non-drug measures have an impact on the cumulative number of confirmed cases in Germany.

**Table 4:** Associations between control policies and cumulative number of confirmed cases in Germany

Parameter	Coefficient	95% CI	P
(Intercept)	11.811	(11.810,11.811)	0.000
Novel Coronavirus Crisis Command	−1.256	(−1.561,-0.951)	0.000
Large gathering cancelled	−0.622	(−0.936,-0.308)	0.000
Real-time COVID-19 risk assessment	−0.729	(−0.889,-0.568)	0.000
The medical emergency plan	−1.703	(− 1.809,-1.596)	0.000
School closure	−1.263	(−1.293,-1.234)	0.000
Restrictions on the import of overseas epidemics	−1.339	(−1.359,-1.319)	0.000
The no-contact protocol	−2.327	(−2.333,-2.320)	0.000

## Discussion

In China, from the end of January to the end of February 2020 is a period of high growth in the number of confirmed cases, and it is also an important stage for the Chinese government to implement non-pharmaceutical interventions. Since March 6, the number of new confirmed cases per day has been less than 100. On April 23, China achieved a single-digit number of new confirmed cases every day, and COVID-19 epidemic has improved significantly. To a certain extent, it has proved that the Chinese government’s decisive and strict non-pharmaceutical measures are remarkable. Since March, the number of confirmed cases in Germany has increased sharply, and the number of daily cases within two weeks has increased from less than 100 to 4000 [8], and the first COVID-19 deaths was reported. On March 12, German states began to close schools, and implemented nationwide stricter non-pharmaceutical interventions. Judged from non-pharmaceutical measures and generalized linear models, the policies of the two countries have both commonalities and differences.

### Differences in non-pharmaceutical interventions between China and Germany

Firstly, China decisively adopted a blocking strategy, and Germany adopted a restriction first and then blocking strategy [9]. There were more than 500 confirmed cases nationwide on January 22, and the Chinese government resolutely adopted a blocking strategy, namely, lockdown Wuhan city. On March 21, Germany became the fifth country with more than 20,000 confirmed cases. The German federal government introduced a “ no-contact protocol “, which means a blocking strategy. Prior to this, the German government has been trying to control the number of new cases within a certain range to reduce the spread of the virus [10].

Secondly, China’s goal is to eliminate the virus, while Germany’s is to protect high-risk groups to reduce social losses. The large number of confirmed cases has led to the dilemma of the health system being on the verge of collapse in Wuhan, China. In more than ten days, Huoshenshan Hospital and Leishenshan Hospital were built, and 16 shelter hospitals were built to classify and intensively care for all patients [11]. Germany focuses on protecting high-risk groups such as monitoring nursing homes, raising hospital beds, increasing medical staff, and focusing its precious medical resources on treating high-risk patients.

### Commonality of non-pharmaceutical interventions between China and Germany

#### Strict lockdown control

In China, as Wuhan, which was the first to identify COVID-19 cases and became the hardest-hit area, the Wuhan New Crown Pneumonia Prevention and Control Headquarters quickly implemented “ lockdown Wuhan”: from 10:00 on January 23, 2020, the city’s bus, subway, ferry, long-distance passenger transport have been suspended. No special reasons, citizens do not leave Wuhan, and the airport, railway station have been temporarily closed [12]. From February 11, all residential areas in the city have been under closed management. The building units where confirmed or suspected patient was located must be strictly controlled [13]. In Hubei Province and throughout the country, the government has basically achieved closed management in urban communities and towns. This played an important role in controlling the source of infection and reducing the spread of COVID-19 across the country.

In Germany, from March to early April, nearly 10 large-scale exhibitions were cancelled or postponed. Religious groups prohibit worship activities. Many professional sports events have been cancelled. Since March 18, all churches, synagogues, mosques, cultural and educational institutions in Baden-Württemberg have been closed. On March 22, the federal government and the state government reached a “no-contact protocol”: people are required to minimize contact with others other than family members, maintain a social distance of 1.5 m in public places, ban restaurant meals, close hair salons and massage parlors, etc. [14] It can be seen from Fig. 2 that a series of blockade measures have an important impact on the gentle trend of the confirmed case curve from late April to the end of May.

#### Improved medical response capabilities

In China, in order to intensively treat critically ill patients and reduce the admission pressure to designated hospitals, Huoshenshan Hospital and Leishenshan Hospital have been built with a total of 2600 beds. From February 5th to March 10th, a total of 16 mobile cabin hospitals were constructed in three batches to offer over 13,000 beds and admitted more than 12,000 patients in Wuhan City [15]. Hubei Province is the hardest hit by COVID-19 in China, so health system is under tremendous pressure. Counterpart assistance can quickly alleviate the conflict between excessive suspected and confirmed cases and insufficient medical personnel, and improve the medical treatment capacity of all cities in Hubei province.

Germany has launched an emergency medical plan including adding more hospital beds and protective equipment, and increasing medical staff [16]. On March 4, the Federal Ministry of Economic Affairs issued a directive prohibiting the export of medical protective materials such as masks, gloves, and protective clothing. The Federal Ministry of Health concentrated on purchasing protective equipment for clinics and hospitals. In order to better take care of the expected increase in patients with COVID-19, the federal government and state governments have reached an agreement in a hospital emergency plan to double the number of intensive care beds in Germany as soon as possible.

## Conclusions

This study confirmed the effectiveness of non-pharmaceutical interventions implemented by China and Germany in the first wave of COVID-19 through quantitative analysis. The two countries have different intervention strategies and control targets: China adopted the blocking strategy, while Germany adopted the restriction and then the blocking strategy. China’s goal is to eliminate the virus, while Germany’s is to protect high-risk groups to reduce social losses. At the same time, the two countries also have similarities in non-pharmaceutical measures: strict blockade is a key means to control the source of infection, and improving medical response capabilities is an important way to reduce the mortality rate.

## Data Availability

No additional data are available.
